# Long Noncoding RNAs: Novel Important Players in Adipocyte Lipid Metabolism and Derivative Diseases

**DOI:** 10.3389/fphys.2021.691824

**Published:** 2021-06-08

**Authors:** Bin Zhang, Saijun Xu, Jinyan Liu, Yong Xie, Sun Xiaobo

**Affiliations:** Institute of Medicinal Plant Development, Peking Union Medical College and Chinese Academy of Medical Sciences, Beijing, China

**Keywords:** long noncoding RNAs, lipid metabolism, adipogenesis, brown/beige adipose, fat, insulin resistance

## Abstract

Obesity, a global public health issue, is characterized by excessive adiposity and is strongly related to some chronic diseases including cardiovascular diseases and diabetes. Extra energy intake-induced adipogenesis involves various transcription factors and long noncoding RNAs (lncRNAs) that control lipogenic mRNA expression. Currently, lncRNAs draw much attention for their contribution to adipogenesis and adipose tissue function. Increasing evidence also manifests the pivotal role of lncRNAs in modulating white, brown, and beige adipose tissue development and affecting the progression of the diseases induced by adipose dysfunction. The aim of this review is to summarize the roles of lncRNAs in adipose tissue development and obesity-caused diseases to provide novel drug targets for the treatment of obesity and metabolic diseases.

## Introduction

Emerging data show that excessive body fat, particularly obesity, is a major risk factor of mortality worldwide (Peeters et al., 2003; Blüher, 2019; Chooi et al., 2019). As the main lipid storage organ, excess adipose tissue is closely related to the occurrence and development of obesity (Sun et al., 2011). When obesity occurs, pathological changes in the morphology, composition, and function of adipose tissues can lead to the occurrence of various metabolic diseases, such as insulin resistance, fatty liver, diabetes, and cardiovascular diseases (Lavie et al., 2009; Blüher, 2019; Ghaben and Scherer, 2019; Schetz et al., 2019). It is of great significance to identify new therapeutic targets for obesity and its related metabolic diseases.

Adipose tissues are physiologically classified into white adipose tissue (WAT) and brown adipose tissue (BAT). WAT is mainly responsible for unnecessary energy storage, whereas BAT functions as fuel oxidation and energy expenditure because of containing abundant mitochondria. With the drug treatments or thermogenic stimuli, WAT possesses the potential to convert into “brown-like” cells. These brite adipocytes can also dissipate energy. Thus, promoting WAT browning might be an effective strategy to prevent obesity.

Recently, many studies have focused on the roles of nonconding RNAs in regulation of adipose tissue activities. Thereinto, long noncoding RNAs (lncRNAs) are defined as long RNA transcripts (>200 bp) not encoding proteins and these lncRNAs are a class of RNA observed to play modulatory roles in many biological processes consistent with their tissue-specificity. LncRNAs are crucial to the regulatory network of adipocyte biology, generating both positive and negative control in lipogenesis and adipogenesis. They also affect adipose tissue functions like white fat browning and brown fat thermogenesis.

This wide range in regulatory roles may make lncRNAs, a promising new therapeutic area in the fight against obesity and related metabolic diseases. However, reviews on the roles of lncRNAs in adipose tissue dysplasia, abnormal lipid metabolism, and associated diseases are very limited. Thus, it is necessary to summarize the latest research progress into the regulation of lncRNAs in lipid metabolism and adipocyte biology. The present review focuses on summarizing the potential of lncRNAs as therapeutic targets for obesity and related diseases caused by lipid metabolism disorders and adipose tissue dysfunction.

## LncRNAs: the Emerging Regulators

### Discovery and Definition of LncRNAs

The development of new technologies, including genome tiling arrays, Global Nuclear Run-On sequencing (GRO-Seq), and Chromatin Isolation by RNA Purification (ChIRP-Seq), helped to identify a mass of new RNAs. LncRNAs are defined as RNA molecules longer than 200 nucleotides (Core et al., 2008; Guttman et al., 2009; Chu et al., 2011). H19, reported in 1990, may be the first identified lncRNA. After transcription by RNA polymerase II, H19 is spliced and polyadenylated like an mRNA, but it encodes no almost protein (Brannan et al., 1990; Bartolomei et al., 1991).

Since the discovery of H19, advances in genome sequencing have identified many more lncRNAs. They generally possess little potential to encode protein for the lack of open reading frames, 3′ untranslated regions, and typical terminal regions (Jarroux et al., 2017), but they play critical roles in a diversity of cellular processes, such as translation, transcription, and epigenetic modification.

### Structure and Function of LncRNAs

**Figure 1:**
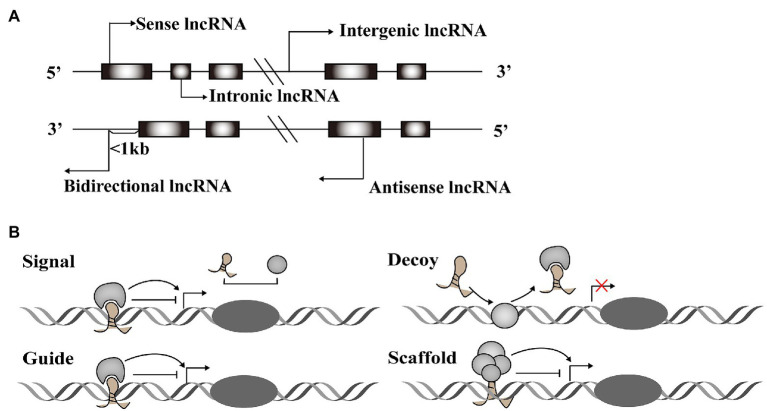
Classification and function modes of Long noncoding RNAs (lncRNAs). **(A)** LncRNAs can be classified into five categories, including sense, antisense, intergenic, intronic, and bidirectional groups according to their relative location with the protein-coding genes. **(B)** LncRNAs function in four archetypes. Archetype I: As signals, lncRNAs can take part in signaling pathways to regulate gene expression. Archetype II: As decoys, lncRNAs combine with transcription factors to block the pathways. Archetype III: As guides, lncRNAs direct protein complexes to specific genome sites. Archetype IV: As scaffolds, lncRNAs recruit several proteins to form ribonucleoprotein complexes.

A vast number of lncRNAs have been identified, but they urgently need to be annotated to be useful in therapeutic applications. Annotation begins by dividing newly discovered lncRNAs into one of five categories based on their relative location in the genome to protein-coding genes (Mattick and Rinn, 2015): sense lncRNAs overlap the nearest protein coding gene along the sense direction; antisense lncRNAs are those whose transcription overlaps an mRNA in any portion; intronic lncRNAs are located in an intron of a protein-coding gene; intergenic lncRNAs that are found between two protein-coding genes; and bidirectional lncRNAs are those whose transcription start site is within 1,000 base pairs (bp) from the neighboring exon and is transcribed in the opposite direction (Figure 1A).

Long noncoding RNAs have diverse structural motifs, including pseudoknots, stem-loops, G-quadruplexes, and triplexes. They mediate gene expression by interacting with DNA and mRNA in the nucleus, or miRNA and protein in the cytoplasm (Willingham et al., 2005; Ulitsky and Bartel, 2013; Sun and Kraus, 2015; Qian et al., 2019). Some lncRNAs act as molecular signals to promote transcription in various metabolic pathways. Others play modulatory roles like decoy, blocking pathways by binding transcription factors. LncRNAs also function as guides, joining with protein complexes and directing them to specific genome sites. These regulatory RNAs can also form scaffolds that recruit modifying enzymes to integrate different signaling pathways (Figure 1B). These four roles are interconnected, and a single lncRNA may exhibit different functions depending on cellular conditions (Wang and Chang, 2011). Taken together, the regulatory role of lncRNAs acts across the whole process of gene expression variability.

### LncRNAs and Diseases

**Figure 2:**
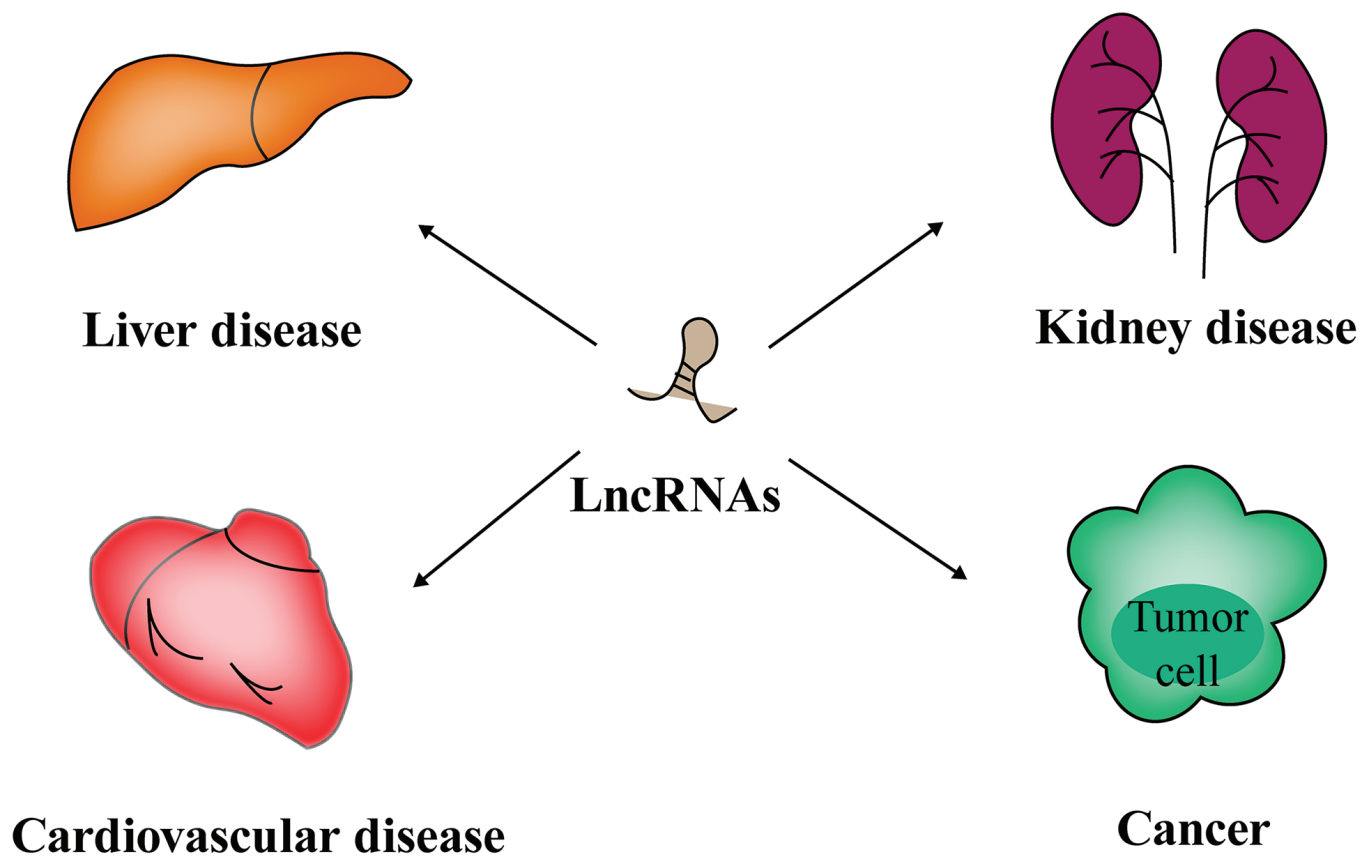
Long noncoding RNAs play critical roles in the pathogenesis of cardiovascular, liver, and kidney disease as well as cancers.

Genome-wide association studies (GWAS) have recognized thousands of single nucleotide polymorphisms (SNPs) from noncoding regions associated with clinical phenotypes, and this intimately links lncRNAs to cardiovascular, liver, and kidney disease as well as some cancers (Figure 2; Table 1; Gao and Wei, 2017; Gong et al., 2018; Hu et al., 2019).

**Table 1:** Key roles of LncRNAs in the diseases.

Disease	LncRNA	Function	References
Cardiovascular disease	Lnc Chaer	Regulates hypertrophy	Wang et al., 2016
	LncRNA Sarrah	Suppresses cardiomyocytes’ apoptosis	Trembinski et al., 2020
	Mhrt	Prevents heart failure	Han et al., 2014
Liver disease	Lnc-LFAR1	Promotes hepatic stellate cell (HSC) activation	Zhang et al., 2017
	Lnc-HSER	Prevents the apoptosis of hepatocytes	Zhang et al., 2019
Kidney disease	Linc-KiAA1737-2, lincMIR210HG	Participates in renal injury response	Lin et al., 2015
	Erbb4-IR	Responsible for renal fibrosis	Feng et al., 2018
	Arid2-IR	Activates the nuclear factor kappa-B (NF-κB) pathway	Zhou et al., 2015
Cancer	DINO	Participates in P53 signal pathway	Schmitt et al., 2016
	TUG1	Promotes the renewal of glioma stem cells (GSCs)	Katsushima et al., 2016
	TROLL-2, TROLL-3	Activates AKT pathway	Napoli et al., 2020

Long noncoding RNAs are variably expressed in the cardiovascular system under different physiological and pathological conditions. For instance, some lncRNAs regulate apoptosis of cardiomyocytes, like lncRNA Sarrah, and hypertrophy, like lncRNA cardiac-hypertrophy-associated epigenetic regulator (Chaer). Some lncRNAs can also reduce the risk of heart failure and acute myocardial infarction. For example, Mhrt prevents Brg1, a chromatin remodeling factor, from binding its DNA targets to prevent heart failure (Han et al., 2014). Another lncRNA, ZFAS1, is a marker of acute myocardial infarction in cardiac systolic function by inhibiting the activity of SERCA2a protein (Zhang Y. et al., 2018). These functions indicate that the roles of lncRNAs are critical to cardiovascular diseases.

High-throughput technologies have also characterized some lncRNAs in liver fibrosis (Bian et al., 2019). Evidence strongly support that lncRNAs are involved in regulating protein-encoding genes in liver fibrosis. For example, Zhang et al. have demonstrated that lncLFAR1 could activate TGFβ and Notch signaling pathways to promote hepatic stellate cell (HSC) activation and liver fibrosis (Zhang et al., 2017). Lnc-HSER, specifically expressed in hepatocytes (HCs), was reported to prevent the apoptosis of hepatocytes through C5AR1-Hippo-YAP pathway and alleviate hepatic fibrosis by inhibiting the HCs epithelial-mesenchymal transition mediated by Notch signaling pathway (Zhang et al., 2019).

Long noncoding RNAs also play vital roles in kidney pathogenesis (Moghaddas Sani et al., 2018). Whole transcriptome profiling analyses identified some lncRNAs associated with acute and chronic renal injury in human proximal renal tubular epithelial cells. Among them, two highlighted lncRNAs, lnc-KiAA1737-2 and lnc-MIR210HG, might participate in renal injury response (Lin et al., 2015). Furthermore, Feng et al. (2018) reported a novel lncRNA Erbb4-IR mediated by transforming growth factor/(TGFβ)/Smad3 responsible for renal fibrosis. Zhou et al. (2015) found Arid2-ir stimulated the nuclear factor kappa-B (NF-κB)- dependent renal inflammation pathway to function in *in vitro* and *in vivo* fibrotic models.

Long noncoding RNAs also participate in the emergence and progression of cancers (Iyengar et al., 2016; Peng et al., 2017). For instance, lncRNA DINO forms a positive feed-back loop with p53 protein to amplify DNA damage signals in the nucleus (Schmitt et al., 2016). Also, activation of the Notch1 signal pathway in glioma stem cells (GSCs) specifically induced expression of the lncRNA TUG1. TUG1 functions to sponge miR-145 in cytoplasm and recruit polycomb in the nucleus to promote the renewal of GSCs (Katsushima et al., 2016). Two TAp63-regulated lncRNAs, TROLL-2, and TROLL-3, can form a trimer complex with the effector WDR26 in cytoplasm to activate AKT pathway (Napoli et al., 2020).

In summary, lncRNAs participate in different disease processes, hinting at their key roles in maintaining homeostasis of human bodies.

## Key Roles of LncRNAs in Controlling Lipid Metabolism and Adipocyte Development

### The Roles of LncRNAs in Controlling White Adipogenesis

**Figure 3:**
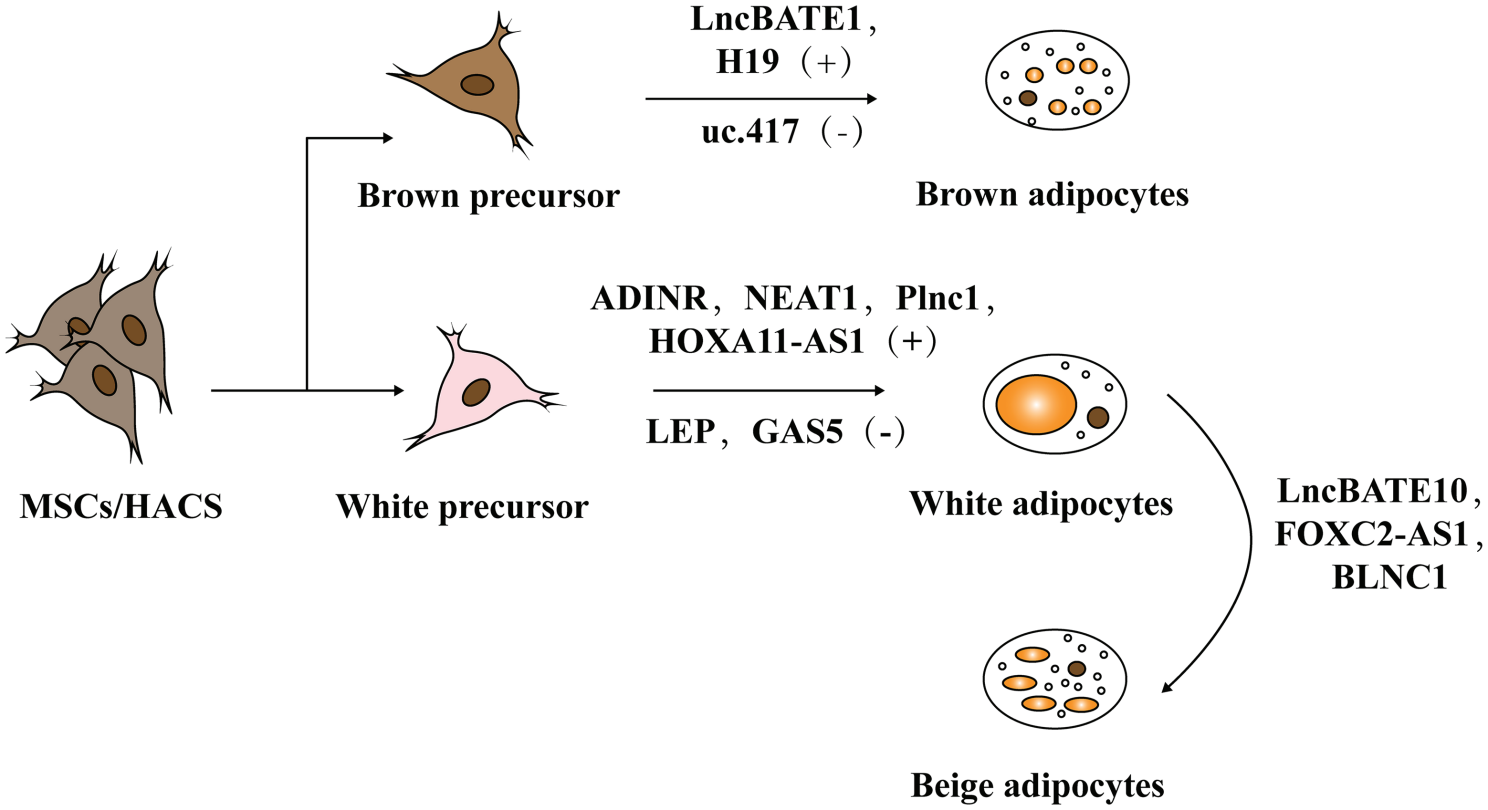
The role of lncRNA in adipogenesis. Adipogenesis mainly includes two stages: mesenchymal stem cells (MSCs) or human adipose tissue-derived stem cells (hASCs) in adipose tissue differentiate into adipose progenitor cells and further into preadipocytes. In the second stage, preadipocytes differentiate into mature adipocytes including white adipose tissue (WAT) and brown adipose tissue (BAT).

Adipogenesis mainly includes two stages. The first stage occurs when embryonic stem cells or mesenchymal stem cells in adipose tissue differentiate into adipose progenitor cells and then to preadipocytes. In the second stage, preadipocytes differentiate into mature adipocytes (Figure 3; Ali et al., 2013; Ambele et al., 2020). The whole process is accompanied by the temporal expression of many crucial adipogenesis-related genes and key transcriptional factors such as lipoprotein lipase (LPL) and sterol regulatory element binding proteins-1c (SREBP-1C). Peroxisome proliferators-activated receptor γ (PPARγ) and CCAAT/enhancer binding proteins (C/EBPs) are common markers of mature adipocytes and the major drivers of adipocyte differentiation. An increasing body of research has found that lncRNAs can regulate these pivotal genes and exert key roles in adipogenesis (Cipolletta et al., 2012; Ghaben and Scherer, 2019).

Human adipose tissue-derived stem cells (hASCs) have the ability to differentiate into both osteocytes and adipocytes. LncRNA MEG3 is upregulated in osteocyte differentiation and downregulated in adipocyte differentiation. Accordingly, MEG3 knockouts promote adipogenic differentiation but suppress osteogenic differentiation, suggesting that MEG3 may serve as a switch for hASCs’ adipogenic or osteogenic differentiation (Li Z. et al., 2017).

Long noncoding RNA ADINR, transcribed from a position about 450 bp upstream of the C/EBPα gene, is induced during adipogenic differentiation. Xiao et al. (2015) revealed that ADINR increases the H3K4me3 methylation but impairs the H3K27me3 histone modification of C/EBPα during adipogenic differentiation to coordinate the transcription of C/EBPα, and this finally promoted adipogenesis in hMSCs. *Plnc1* was an lncRNA transcribed from a position 25,000 bp upstream of PPARγ2. *In vitro* studies showed that a plnc1 knockout decreases the expression of PPARγ, C/EBPα, and Fatty Acid Binding Protein 4 (AP2), which subsequently suppresses the differentiation of ST2 cells and BMSCs into mature adipocytes. However, overexpression of *Plnc1* has the opposite effect. Mechanistically, plnc1 enhances the transcriptional activity of PPARγ2 by decreasing the methylation level of CPG in PPARγ2 promoter (Zhu et al., 2019).

Long noncoding RNA MIR-140 knockout decreases the expression of key transcription factors (C/EBP and PPARγ), which directly impairs the mice’s adipogenic capabilities. It suggestd that MIR-140 is a necessary regulatory factor for adipocyte differentiation. Non-coding RNA qPCR array showed that NEAT1 is highly conserved between humans and mice and is upregulated in hASC differentiation. Using RNA hybrid, Gernapudi et al. (2016) identified the miR140 binding site in NEAT1 and they found that mature miR-140 can physically interact with NEAT1 in the nucleus. Further experimental data indicated that the binding of MIR-140 to NEAT1 in the nucleus increases NEAT1 stability, thus promoting adipogenesis.

Nuermaimaiti et al. (2018) found HOXA11-AS1 knockdown inhibits the transcription of adipogenesis-related genes in hASCs differentiation model, leading to the suppression of adipogenic differentiation and alleviation of lipid accumulation. Moreover, HOXA11-AS1 is highly expressed during the process of adipocyte differentiation in obese patients, indicating that it might be a potential target for the treatment of obesity.

In addition, bioinformatics analyses were used to identify a series of key mRNAs, miRNAs, and lncRNAs in hASCs adipogenesis. Several interaction axes were observed to regulate the adipogenic differentiation of hASCs, among which the leptin (LEP)-related axis was particularly important by analyzing the region upstream and downstream of leptin gene (Guo and Cao, 2019).

Some lncRNAs are reported to be potential targets that inhibit adipogenesis. The investigation based on a cDNA chip to analyze the adipogenesis regulatory genes found that the expression of lnc-U90926 was negatively correlated with the differentiation of 3 T3-L1 preadipocyte. Then, using RNA fluorescence *in situ* hybridization (FISH), researchers confirmed lnc-U90926 mainly localized to the cytoplasm of mice’s preadipocytes. Gain- and loss-of-function experiments showed that the overexpression of lnc-U90926 blocked adipocyte differentiation in 3 T3-L1 as evidenced by reductions in lipid accumulation and specific protein expression, like that of PPARγ and AP2. Additionally, lnc-U90926 had lower expression levels in obese mice, which indicates it can inhibit adipogenesis by suppressing the transcriptional activity of key genes (Chen et al., 2017).

In a study on adipogenic differentiation, Li M. et al. (2018) found lncRNA GAS5 is negatively associated with adipogenesis of mesenchymal stromal cells (MSCs). Using luciferase reporter assays they further discovered that GAS5 inhibits MSCs’ adipogenic differentiation by competitively sponging miR-18a. Likewise, Liu et al. (2018) determined that GAS5 has a negative role in 3 T3 cells’ adipogenesis by repressing miR-21a-5p. Thus, GAS5 is an important regulator in the adipogenic differentiation.

LncHCG11 is another target for inhibiting adipogenesis. Li et al. (2020) found that the expression of lncHCG11 declines during the adipogenic differentiation in an *in vitro* hADMSCs differentiation model. Specifically, both the activity of related lipogenesis enzymes and the expressions of adipogenic proteins increase in HCG11 knockdowns, while the reverse response is observed when HCG11 is overexpressed. Bioinformatics analyses of the HCG11/miR204-5p/SIRT1 axis, in addition to experimental evidence, show that when co-transfected with a miR-204-5p mimic and pcDNA-HCG11, the miR-204-5p mimic reduced SIRT1’s inhibitory effects on the expression of lipogenesis enzymes and adipogenic marker proteins to reverse pcDNA-HCG11’s depression effects on adipogenesis.

The majority of lncRNAs are poorly conserved among mammals, many therapeutic applications necessitate that more attention is placed on identifying and characterizing lncRNAs in human adipose tissue. Zhang X. et al. (2018) performed RNA-seq on subcutaneous biopsy samples from healthy, lean humans and detected 1,001 adipose-enriched lncRNAs, among which lnc-ADAL is the most highly expressed. Lnc-ADAL is a non-conserved lncRNA closely tied to obesity. ShRNA-mediated knockouts suppressed the expression of lipid synthesis genes, while ASO-mediated knockouts not only impaired the expression of lipid synthesis genes in mature adipocytes but also damaged the preadipocyte differentiation. Researchers then verified that lnc-ADAL interacted with both the nuclear protein hnRNPU and cytoplasmic protein IGF2BP2 to control preadipocyte differentiation and *de novo* lipogenesis. These studies collectively support that lncRNAs emerge as important regulatory players in the process of white adipogenesis (Table 2).

**Table 2:** Key roles of LncRNAs in regulating white adipogenesis.

LncRNA	Roles in adipogenesis	Proposed mechanism of action	References
MEG3	Negative	Inhibits adipogenesis *via* downregulation of miR-140-5p	Li Z. et al., 2017
ADINR	Positive	Activates C/enhancer binding proteins (EBPs) transcription	Xiao et al., 2015
Plnc1	Positive	Increases PPAR-γ2 promoter activity	Zhu et al., 2019
NEAT1	Positive	Mature miR-140 binds to NEAT1 to increase NEAT1 expression	Gernapudi et al., 2016
HOXA11-AS1	Positive	Promotes adipogenic-related genes’ transcription (CEBP-α, DGAT2, etc.)	Nuermaimaiti et al., 2018
LncRNA-LEP	Positive	Activation of RP11-552F3.9- hsa-miR-130b-5p- LEP interaction axes increases leptin expression	Guo and Cao, 2019
GAS5	Negative	Suppression of miR-18a decreases CTGF expression	Li M. et al., 2018; Liu et al., 2018
Linc-ADAL	Both	Interacts with hnRNPU and IGF2BP2	Zhang X. et al., 2018

### The Roles of LncRNAs in Brown/Beige Fat Development and Their Function

Brown adipocytes (BAT) possess abundant mitochondria in the cytoplasm and high levels of uncoupling protein 1 (UCP1; Cannon et al., 1982). Accordingly, they can generate heat through uncoupling the lipid oxidative phosphorylation, facilitating the burning of fatty acid and glucose. Earlier studies supported that BAT is present and active in newborns to maintain their body temperature through non-shivering thermogenesis (Cannon and Nedergaard, 2004). Positron emission tomography (PET) detected considerable amounts of BAT in adult males, suggesting that BAT also plays an essential role in adult metabolism (Nedergaard et al., 2007). To date, a series of studies have indicated that lncRNAs are indispensable regulators in brown adipogenesis and thermogenesis (You et al., 2015).

For example, H19, a maternally inherited lncRNA, is inversely correlated with Body Mass Index (BMI) in humans. Schmidt et al. (2018) reported that H19 overexpression promotes adipogenesis and mitochondrial respiration in BAT by recruiting PEG-inactivating H19-MBD1 complexes. This study illustrated the function of H19 in regulating the BAT thermogenic gene program and metabolism.

Cui et al. (2016) identified uc.417, an ultra-conserved lncRNA that is abundant in differentiated brown adipocytes. They found that overexpression of uc.417 inhibits the differentiation of brown fat cells. They also analyzed oxygen consumption of brown adipocytes with uc.417 overexpressed and evaluated the negative roles of uc.417 overexpression in BAT’s thermogenesis progress. However, knockouts of uc.417 had no significant impact on the differentiation and thermogenesis of brown adipocytes. Another lncRNA, lncBATE1, has been found to interact with hnRNP U that is necessary for brown adipogenesis and maintaining its thermogenic capacity (Alvarez-Dominguez et al., 2015). These data strongly support lncRNAs’ roles in driving brown fat formation and maintaining energy balance.

Beige fat is usually stored in white fat warehouses and can be differentiated into specific beige precursor cells in WAT or derived directly from the browning of mature white fat cells under exposure to cold or other stimuli. Because they can highly express UCP1 protein and function as BAT, induction of beige fat adipogenesis helps resist obesity and is assumed to be a promising strategy to covert unhealthy WAT into metabolically active BAT (Guerra et al., 1998; Bartelt and Heeren, 2014; Wang and Seale, 2016).

Wang Y. et al. (2020) found high expression of FOXC2-AS1 in the forskolin (Fsk)-stimulating adipocytes with high levels of UCP1 and peroxisome proliferator-activated receptorγcoactivator-1α (PGC1α). They found that FOXC2-AS1 may promote WAT browning and thermogenic program through the autophagy signaling pathway. The result showed that lncRNAs also play crucial roles in the development and functional activation of beige adipose.

In fact, some lncRNAs regulate through the co-expression network. For instance, Bai et al. (2017) found a large number of lncRNAs embedded into metabolic pathways by establishing an mRNA-lncRNA co-expression network. Through this network, they identified lncBATE10 that is rich in BAT and can decoy Celf1 from Pgc1α, activating Pgc1α expression and promoting thermogenesis and WAT browning.

Additionally, AK079912 is another BAT-enriched lncRNA. Knockdown of AK079912 decreases the expression of vital adipogenic and thermogenic genes; while overexpression upregulates the thermogenic program by increasing protein expressions of UCP1 and mitochondria electron transport chain (ETC) complexes. Moreover, Xiong et al. (2018) found expression of AK079912 in inguinal WAT could induce their browning.

Blnc1 is rich in both the brown and beige adipocytes. Over-expression of BLNC1 in brown adipocytes increases the expression of thermogenic genes through the formation of the Blnc1/hnRNPU/EBF2 ribonucleoprotein complex (Mi et al., 2017). The effects of Blnc1 on beige adipocytes were also evaluated. During brown fat whitening induced by a high-fat diet (HFD), specific deactivation of Blnc1 in the fat tissue not only accelerated the BAT to bleach, but also exacerbated the inflammation. However, fat-specific Blnc1 transgenic mice have the opposite effects. The molecular mechanism is that Blnc1, as a target of EBF2, built a feedforward regulatory loop to promote browning of WAT (Zhao et al., 2014). Additionally, Lin et al. (2015) found that BTB domain-containing 7b (Zbtb7b) could recruit the lncRNA Blnc1 through hnRNP U to increase thermogenic genes expression, and the function of Blnc1 is conserved in mice and humans (Li S. et al., 2017).

Thus, lncRNAs are important regulators for activating brown/beige adipocytes to function with the benefit of decreasing serum triglycerides and fighting against obesity (Table 3).

**Table 3:** Key roles of LncRNAs in regulating brown/beige adipogenesis.

LncRNA	Roles in BAT or beige adipocytes	Proposed mechanism of action	References
H19	Positive	Recruits PEG-inactivating H19-MBD1 complexes to control brown adipocyte differentiation	Schmidt et al., 2018
uc.417	Negative	Suppresses p38MAPK’s phosphorylation to impair adipogenesis	Cui et al., 2016
LncBATE1	Positive	Binds to hnRNP U to facilitate brown adipogenesis	Alvarez-Dominguez et al., 2015
FOXC2-AS1	Positive	Promote white-to-brown adipocyte conversion through autophagy activation	Wang Y. et al., 2020
LncBATE10	Positive	Elevate Pgc1α expression through decoying Celf1 from it	Bai et al., 2017
BLNC1	Positive	Form Blnc1/hnRNPU/EBF2/Zbtb7b ribonucleoprotein complexes to accelerate white adipose tissue (WAT) browning	Zhao et al., 2014; Li S. et al., 2017; Mi et al., 2017

### LncRNA and Lipid Homeostasis in the Liver

Besides the known roles in adipocytes, a series of studies have shown that lncRNAs regulate lipid metabolism in the liver by targeting several crucial transcription factors, such as liver X receptor (LXRs), sterol-regulatory element binding proteins (SREBPs), and peroxisome proliferation-activated receptor α (PPARα). These transcription factors are regulators of gene expression for the synthesis and uptake of cholesterol, fatty acids, and phospholipids in the liver (Sun and Lin, 2019). Here, we summarize the regulatory mechanisms of lncRNAs in liver lipid homeostasis (Table 4).

**Table 4:** Key roles of LncRNAs in regulating lipid homeostasis in the liver.

LncRNA	Tissue/cell type	Loss-of-Function	Gain-of-function	References
LncHR1	Hepatic cells	-	Decreases triglycerides and lipid droplets	Li D. et al., 2017
LncLSTR	Human liver	Reduces triglyceride levels	-	Li et al., 2015
Lnc19959.2	Rat liver	Lowers plasma triglyceride	-	Wang J. et al., 2020
DYN-LRB2-2	THP-1 and Raw264.7 cells	-	Upregulates cholesterol efflux	Li Y. et al., 2018
TUG1	NCTC 1469 cells	-	Slows down CE rate	Yang and Li, 2020
LncARSR	Hepatic cells	-	Promotes cholesterol biosynthesis	Huang et al., 2018
HULC	HCC cells	-	-	Cui et al., 2015
Lnc-HC	Rat liver	Increases lipid accumulation	-	Lan et al., 2019

LncHR1, identified in human hepatoma cells infected with HCV, negatively regulates the expression of SREBP-1c and fatty acid synthase. Overexpression of lncHR1 inhibits the accumulation of triglyceride and lipid droplets in liver cells (Li D. et al., 2017). Li et al. (2015) found liver-enriched lncLSTR could decrease the plasma triglyceride levels (TDP43/FXR/APOC2) by competitively binding with TDP-43 to regulate the expression of Cyp8b1, leading to the activation of Apoc2 *via* the nuclear receptor farnesoid-X-receptor (FXR) pathway.

Additionally, Wang J. et al. (2020) found that Lnc19959.2 was upregulated in high fat-induced hepatocytes. Mechanically, overexpressed lnc19959.2 promotes the expression of ApoA4 by interacting with nuclear protein Purb. Lnc19959.2 specifically binds to hnRNPA2B1 to negatively regulate the expression of genes related to TG metabolism. Taken together, the investigations of lncHR1 and lnc19959.2 indicate that lncRNAs specifically expressed in liver are emerging as key players in the regulation of triglycerides.

Long noncoding RNAs are also closely related to the cholesterol metabolism. Hu et al. (2019) found that ox-LDL could significantly upregulate the expression of lincRNA-DYN-LRB2-2, directly leading to an increased expression of ATP-binding cassette transporter A1 (ABCA1). Elevated expression of ABCA1 mediated cholesterol efflux (CE) in foam cells, thus reducing cholesterol levels (Li Y. et al., 2018). Another study found that lncTUG1 inhibited CE by inhibiting the expression of APOM in an miR-92a/FXR1 dependent manner (Yang and Li, 2020). Additionally, Huang et al. (2018) found overexpression of lncARSR can activate PI3K/Akt signal pathway, promoting the expression of transcription factor SREBP2. This transcription factor, in turn, increased the expression level of the rate-liming enzyme in cholesterol, HMG-CoA reductase (HMGCR), and accelerated cholesterol biosynthesis in liver.

Another group of lncRNAs can influence both the cholesterol and triglyceride level. HULC was reported to mediate abnormal lipid metabolism in hepatocellular carcinoma and elevate the levels of intracellular triglycerides and cholesterol by activating ACSL1/miR-9/PPARA signaling pathway (Cui et al., 2015). Moreover, Lan et al. (2019) identified a novel noncoding RNA lnc-HC that not only reduced cholesterol efflux by inhibiting the expression of cholesterol 7α-hydroxylase (Cyp7a1) and ABCA1, but also promoted hepatic triglyceride metabolism by negatively regulating PPARγ expression. Overall, lncRNAs play vital roles in maintaining lipid homeostasis in the liver and emerge as important targets to alleviate liver diseases caused by fat metabolic disorders, such as nonalcoholic fatty liver disease.

## The Potential of Lncrnas As Therapeutic Targets of Related Diseases Induced By Adipocyte Dysfunction

Obesity is becoming a global pandemic and usually leads to some metabolic diseases, including nonalcoholic fatty liver disease (NAFLD), diabetes, and other diabetic complications (Kusminski et al., 2009). However, therapeutic targets and methods for the treatment of obesity and related metabolic diseases remain limited in the clinic (Kakkar and Dahiya, 2015). The existing approved drugs mainly function to combat obesity by reducing intestinal fat absorption or suppressing appetite (Tsilingiris et al., 2020). Treatments to improve related metabolic disease are mainly through the use of drugs with hypoglycemic, anti-hypertensive or lipid-lowering activity. However, these agents usually have larger side effects on human body and their protective effect is limited. For examples, statins commonly cause muscular adverse reactions such as fatal rhabdomyolysis (Bouitbir et al., 2020). Chronic administration of niacin can decrease glucose tolerance and increase uric acid level, potentially induce gouty attacks and impaired liver function (Kei et al., 2011). Thus, there is an urgent need to identify novel targets and develop new effective and safe drug candidates. Functional studies of lncRNAs provide new insight for the establishment of related drugs.

### The Roles of LncRNAs in Insulin Resistance

Low-grade chronic inflammation, as a marker of obesity, has been identified as a vital risk factor for the occurrence of insulin resistance (Glass and Olefsky, 2012). The increased free fatty acids in the obese can promote NF-κB signaling, which upregulates the expression of pro-inflammatory cytokines, such as TNF-α and IL-6 (Tangvarasittichai, 2015). The pro-inflammatory signals then inhibit the function of insulin in metabolic tissue, thereby mediating insulin resistance.

Because the inflammation reaction is a phenotype caused by macrophages responding to excessive lipid accumulation, researchers have focused on macrophages to reduce this inflammation. These studies have found several novel lncRNAs enriched in macrophages and differentially expressed in diet-induced mice models with obesity and early diabetes. For example, Stapleton et al. (2020) found that MIST was associated with a macrophage anti-inflammatory phenotype during gain- and loss-of-function experiments. When transcription or interaction with RNAs of MIST was disrupted, expression of inflammatory genes heavily increased. They then discovered that MIST interacted with poly ADP-ribose polymerase (PARP1) in the nucleus. It may act as a protective lncRNA by interfering with the formation of pro-inflammatory cytokines that are closely correlated with insulin sensitivity index. In addition, Zhang et al. (2020) identified lncRNA uc.333 that improved obesity-induced insulin resistance by binding to miR-223. Moreover, Liu S. et al. (2014) reported that knocking out lncRNA SRA protected mice against high fat diet-induced obesity and improved their glucose tolerance. All the above indicate that lncRNAs are potential therapeutic targets for improving insulin resistance.

### The Roles of LncRNAs in Hepatic Steatosis

Metabolic disorders often cause non-alcoholic fatty liver disease (NAFLD) and hepatic steatosis (NASH) that are characterized by the accumulation of liver lipids (Kawano and Cohen, 2013). If NAFLD is not treated in time, it can gradually develop into NASH and then into hepatocellular cancer or other malignant diseases (Kanwal et al., 2018; Lindenmeyer and McCullough, 2018). LncRNAs may be checkpoints to enable unhealthy hepatic lipogenesis and impair liver lipid homeostasis. In an animal model with NAFLD, Wang (2018) found that lncRNA-NEAT1 knockdown may alleviate the NAFLD *via* regulating the mTOR/S6K1 signaling pathway. Another lncRNA, MALAT1, is highly expressed in livers of ob/ob mice. Mechanism research demonstrated that inhibiting MALAT1 suppresses hepatic lipid accumulation and attenuates hepatic steatosis by reducing the stability of nuclear SREBP-1c protein in hepatocytes (Yan et al., 2016). Considering that hepatic expression of BLNC1 is evidently elevated in the obesity and NAFLD in mice, researchers studied the effects of BLNC1 on HFD – induced hepatic steatosis. They found that BlNC1 deficiency greatly inhibits both the plasma TAG levels and the induction of SREBP1 protein expression by LXR agonists. Additionally, liver-specific BLNC1 knockout mice exhibited resistance to HFD-induced hepatic steatosis, manifested as reduced hepatic damage and fibrosis. These responses indicated that BLNC1 may work cooperatively with LXR to control hepatic lipid metabolism, which may serve as a therapeutic target for the treatment of NAFLD patients (Zhao et al., 2018).

### The Roles of LncRNAs in Atherosclerosis

The pathogenesis of atherosclerosis is complex, although current research as established that dyslipidemia (abnormal plasma cholesterol and lipoprotein levels) is one of the crucial risk factors (Gisterå and Ketelhuth, 2018). Since many studies have confirmed lncRNAs’ regulatory roles in lipid homeostasis, some researchers think they may take part in the development of atherosclerosis. LncRNA KCNQ1OT1 could inhibit cholesterol efflux and promote lipid accumulation in macrophages *via* the miR-452-3p/HDAC3/ABCA1 pathway, and, thus, contribute to the development of atherosclerosis (Yu et al., 2020). A key node in atherosclerosis is when macrophages uptake lipoproteins and form foam cells (Tabas and Bornfeldt, 2020). Kanwal et al. (2018) found lncRNA E330013P06 upregulated the expression of CD36 in macrophages to promote foam cells formation (Reddy et al., 2014). Another lncRNA, AT102202, controls the expression of mRNA-3-hydroxy-3-methylglutaryl coenzyme A reductase (HMGCR) to affect the accumulation of cholesterol in macrophages (Liu et al., 2015). Although the function and mechanism of lncRNAs in atherosclerosis still need further exploration, lncRNAs are apparently vital regulatory factors implicated in the pathological process of atherosclerosis. LncRNAs have may have future clinical applications as biomarkers and potential therapeutic targets of atherosclerosis.

### The Roles of LncRNAs in Diabetic Complications

**Figure 4:**
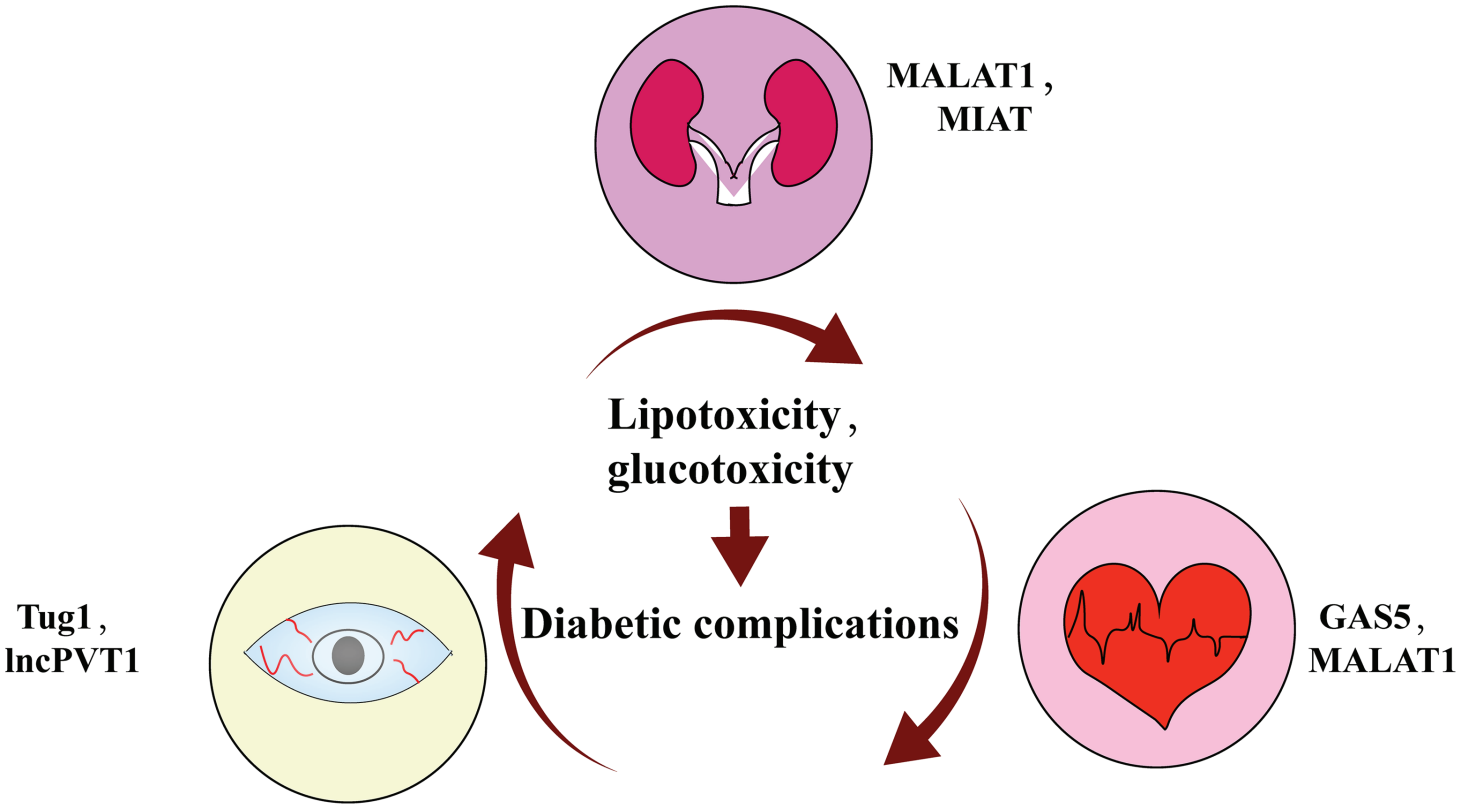
Summary of key roles of lncRNAs in diabetic complications.

Epidemiological studies have reported a large increase in the prevalence of diabetes, which mainly happens among people with long-term abdominal obesity (Ampofo and Boateng, 2020). The main harm of diabetes lies in its severe complications, such as diabetic retinopathy, nephropathy, cardiomyopathy, etc.; however, the therapeutic targets and drugs still remain limited (Harding et al., 2019). LncRNAs recently gained attention for their regulatory roles in diabetic complications (Figure 4).

Diabetic retinopathy is a common complication caused by hyperglycemia and dyslipidemia. At present, retinal gene expression profiles have identified more than 300 differentially expressed lncRNAs associated with diabetic retinopathy (Yan et al., 2014). One study showed that an lncRNA MALAT1 knockdown can inhibit the proliferation, migration, and tube formation of retinal endothelial cells (Liu J.-Y. et al., 2014). Radhakrishnan and Kowluru (2021) verified MALAT1 suppression attenuates oxidative damage *via* the Keap1-Nrf2 pathway to improve retinal vascular function and slow diabetic retinopathy. Additionally, Yan et al. found lncRNA MIAT can form a feedback loop with VEGF and miR-150-5p to regulate endothelial cell function and improve the microvascular dysfunction induced by diabetes (Yan et al., 2015). Thus, lncRNAs are involved in the development of diabetic retinopathy and may be potential therapeutic targets for the disease.

Chronic hyperglycemia and dyslipidemia are also the main causes of diabetic nephropathy, a microvascular complication characterized by the damage of glomerular capillaries (Opazo-Ríos et al., 2020). Accumulating evidence supports lncRNAs’ involvement in the occurrence and development of this disease.

For example, LncRNA Tug1 was found to interact with PGC-1 to regulate its expression and affect the mitochondrial bioenergetics in podocytes (Long et al., 2016). Additionally, in type II diabetic patients with end-stage renal disease, lncPVT1 controlled the accumulation of the extracellular matrix and the progression of renal cells fibrosis, thereby mediating the development of diabetic kidney disease (Alvarez and DiStefano, 2011).

Diabetic cardiomyopathy is mainly caused by cardiac lipotoxicity (Nakamura and Sadoshima, 2020). LncRNA GAS5 regulates the miR-34b-3p/AHR pathway to repress the pyroptosis induced by NLRP3 inflammasome activation, making GAS5 a potential therapeutic target (Xu et al., 2020). Wang et al. (2021) found that silencing lncRNA MALAT1 could inhibit EZH2 expression *via* the EZH2/miR-22/ABCA1 signaling axis, which prevents cardiomyocyte apoptosis and attenuates cardiac dysfunction. Above all, many studies have demonstrated that lncRNAs play functional roles in the pathological processes of diabetic complications and have potential therapeutic significance for the diseases.

## Conclusion

This review mainly summarized the studies on the regulation of lncRNAs in lipid metabolism in the liver as well as the development and function of adipose tissue. Meanwhile, numerous examples have been provided of a series of lncRNAs involved in adipocyte dysfunction-induced diseases, such as insulin resistance, hepatic steatosis, and diabetic complications. Currently, most of the drugs used to fight obesity target proteins, but these drugs have side effects because of the unintended regulation of non-target protein. Thus, the development of new drugs that target nucleic acids might provide a novel therapeutic strategy for the treatment of obesity and its related diseases.

Some targeted nucleic acid therapies, such as antibacterial and anticancer therapy are gradually being applied to treat some diseases (Aradi et al., 2020; Javanmard et al., 2020). Nucleic acid targeting methods are considered the third generation of therapeutic drugs, and three main strategies have been reported thus far: (i) small interfering RNA (siRNA), targeting cytoplasmic lncRNAs, like Givlaari (Givosiran) which was approved to treat adult acute hepatic porphyria; (ii) antisense oligonucleotides (ASO), targeting nuclear lncRNAs. LNA gapmeR ASO-targeting lncRNA MALAT1 possesses anti-multiple myeloma activity (Amodio et al., 2018); (iii) CRISPR/Cas9 technology is suitable for dual-located lncRNAs, and it has been widely used in the discovery and annotation of lncRNAs but, as of yet, is not ideal for systematic drug delivery. These three therapies have been approved for clinical application, but all face off-target problems (Caffrey et al., 2011; Fu et al., 2014; Rinaldi and Wood, 2018).

Some lncRNAs positively regulate white adipogenesis and are upregulated in the obese patients, and they might be suitable ASO targets. However, no lncRNA targeting drugs have entered clinical trials, and many therapies are still in the preclinical stage. Because many lncRNAs are poorly conserved, researchers often struggle to transfer successful mouse model experiments to human treatments. Thus, more humanized lncRNAs remain to be probed and more applicable preclinical study models need to be established. On the other hand, the regulation network of lncRNAs is complex and it is not easy to achieve accurate regulation *in vivo*. Accordingly, it is necessary to establish a highly organized lncRNA research database, D-LNC platform is such an attempt to query and analyze the modification effects of drugs on the expression of lncRNAs (Jiang et al., 2019).

To sum up, the study of lncRNAs in adipose metabolism and obesity-caused diseases, as well as the therapeutic strategies presented above, may provide novel medication for the treatment of obesity and related metabolic diseases.

## Author Contributions

BZ, YX, and SuX contributed to the conception of the review. BZ and SaX contributed significantly to the complete manuscript preparation. SaX and JL contributed to constructive discussions. All authors contributed to the article and approved the submitted version.

### Conflict of Interest

The authors declare that the research was conducted in the absence of any commercial or financial relationships that could be construed as a potential conflict of interest.
